# Benchmarking of 16S rRNA gene databases using known strain sequences

**DOI:** 10.6026/97320630017377

**Published:** 2021-03-31

**Authors:** Kunal Dixit, Dimple Davray, Diptaraj Chaudhari, Pratik Kadam, Rudresh Kshirsagar, Yogesh Shouche, Dhiraj Dhotre, Sunil D Saroj

**Affiliations:** 1 Symbiosis School of Biological Sciences (SSBS), Symbiosis International (Deemed University), Pune, India; 2 National Center for Microbial Resource (NCMR), National Center for Cell Science (NCCS), Pune, India; 3 Reliance Life Sciences Pvt Ltd, Rabale, Mumbai, India

**Keywords:** 16S rRNA gene, Genomic Databases, Taxonomic Discrepancy, QIIME

## Abstract

16S rRNA gene analysis is the most convenient and robust method for microbiome studies. Inaccurate taxonomic assignment of bacterial strains could have deleterious effects as all downstream analyses rely heavily on the accurate assessment of microbial taxonomy. The use of mock communities to check the reliability of the results has been suggested. However, often the mock communities used in most of the studies represent only a small fraction of taxa and are used mostly as validation of sequencing run to estimate sequencing artifacts. Moreover, a large number of databases and tools available for classification and taxonomic assignment of the 16S rRNA gene make it challenging to select the best-suited method for a particular dataset. In the present study, we used authentic and validly published 16S rRNA gene type strain sequences (full length, V3-V4 region) and analyzed them using a widely used QIIME pipeline along with different parameters of OTU clustering and QIIME compatible databases. Data Analysis Measures (DAM) revealed a high discrepancy in ratifying the taxonomy at different taxonomic hierarchies. Beta diversity analysis showed clear segregation of different DAMs. Limited differences were observed in reference data set analysis using partial (V3-V4) and full-length 16S rRNA gene sequences, which signify the reliability of partial 16S rRNA gene sequences in microbiome studies. Our analysis also highlights common discrepancies observed at various taxonomic levels using various methods and databases.

## Background

Next-Generation Sequencing (NGS) techniques are capable of generating high quality, comparable data [1]. Traditional methods are left behind as sequencing of 16S rRNA gene amplicons is now a well-established robust method for bacterial identification. The method has revolutionized the microbiome research as well as the field of medicine and pathology. 16S rRNA gene sequencing has been used to decipher the human microbiome associated with various disorders, including colorectal cancer [2]. Moreover, the method has been proven to be quick and reliable in diagnostic laboratories for pathogen identification [3,4]. Although 16S rRNA gene based analysis remains to be the gold standard, proper precautions need to be taken during sequencing, preprocessing of data, and subsequent downstream analyses. The selection of variable region, choice of method for OTU clustering, selection of reference databases, and sequencing platform has been shown to play an important role in the assessment of microbial diversity [5,6]. Although V3-V4 or only V4 region is used widely by the scientific community, the choice of the region has been observed to affect community identification [7,8]. Also, it is known that sequencing errors with different sequencing platforms could reduce the reliability of the analysis [9]. Another limitation is that the taxonomic assignment is dependent on the reference database. Accuracy and resolution of taxonomic assignments might differ depending upon the quality and quantity of reference databases [10]. Genomic data for the 16S rRNA gene of bacterial type strains are pooled and preserved in several databases like Genomic-based 16S ribosomal RNA gene Database (GRD), SILVA, Ribosomal Database Project (RDP) etc. Different pipelines like Quantitative Insights Into Microbial Ecology (QIIME) [11], Mothur [12], Metagenomic Rapid Annotations using Subsystems Technology (MG-RAST)[13] etc. are used worldwide for high precision and quick analysis. Different methods are used to overcome the limitations regarding 16S rRNA gene analysis. One of them is the use of training datasets, which have an impact on the classification and robustness of analysis of big datasets of bacterial 16S rRNA gene [14]. Furthermore, the use of mock communities has been suggested to check the reliability of the sequencing output. Several laboratory specific or commercially made mock microbial communities like 'ATCC Gut microbiome whole cell mix', 'ATCC Vaginal microbiome whole cell mix' (Source- https://www.atcc.org) have proven to be effective in method optimization as well as to achieve transparency across different studies [15, 16]. However, though mock microbial communities serve the purpose of estimating sequencing errors, they mostly represent minimal diversity. They thus cannot be used as a standard for taxonomic identification by analysis pipeline and databases. Thus it is a necessity to have a 16S rRNA gene analysis pipeline validated using a standard data set with known taxonomic identification. Several earlier studies have compared different databases and analysis pipelines like one by Nilakanta et al., which reviewed the existing analysis pipelines for genomic data analysis and suggested that Mothur and QIIME are the two outstanding pipelines due to their comprehensive features and support documentation. The limitation of the study was that they did not use a unified dataset to compare the performance of pipelines in terms of taxonomic assignment [17]. Another study by Erica Plummer et al., used a 35 infent fecal samples for comparison of three widely used analysis methods QIIME, Mothur, and MG-RAST, for accuracy in taxonomic assignments using only V3-V5 hypervariable regions of 16S rRNA gene [18]. In the present study, we used authentic and validly published, type strain, full length (1200-1500bp) and partial (In silico extracted V3-V4 region) 16S rRNA gene sequences (n=5895). These sequences were compared against various databases with QIIME pipeline, which incorporate various algorithms for quality control, clustering similar sequences, assigning taxonomy, calculating diversity measures and visualizing. We used 16S rRNA gene sequences of type strains obtained from the RDP database as it allows the option to download the bulk dataset [19]. Three different databases were used for microbiome analysis, namely Greengenes, SILVA, and EzTaxon [20] which used for 16S rRNA gene-based microbiome studies [21-26]. Although several different analysis pipeline alternatives like Dada2, QIIME 2, deblur are currently avalilable, we chose QIIME 1 pipeline as it is well accepted by scientific community having over 22000 citations and being used globally for microbiome analysis [27-36].

## Methodology

### Analysis of the sample Data:

Type strain 16S rRNA gene sequences from the RDP website (https://rdp.cme.msu.edu/) of full length (length < 1200bp) 16S rRNA gene sequences (n=5,895) were downloaded along with their seven (species) level taxonomy. Publically available databases viz. SILVA (128 release), Greengenes, and EzTaxon were used for the OTU (Operational Taxonomic Unit) clustering and taxonomic assignment [37]. In this study, we used the most cited and widely used databases only. Full database of 97% and 99% identity threshold were downloaded from SILVA, Greengenes, and EzTaxon websites. Both 16S rRNA gene sequences, along with the corresponding taxonomy, was available in all databases.

### Methodology:

**Figure 1:**
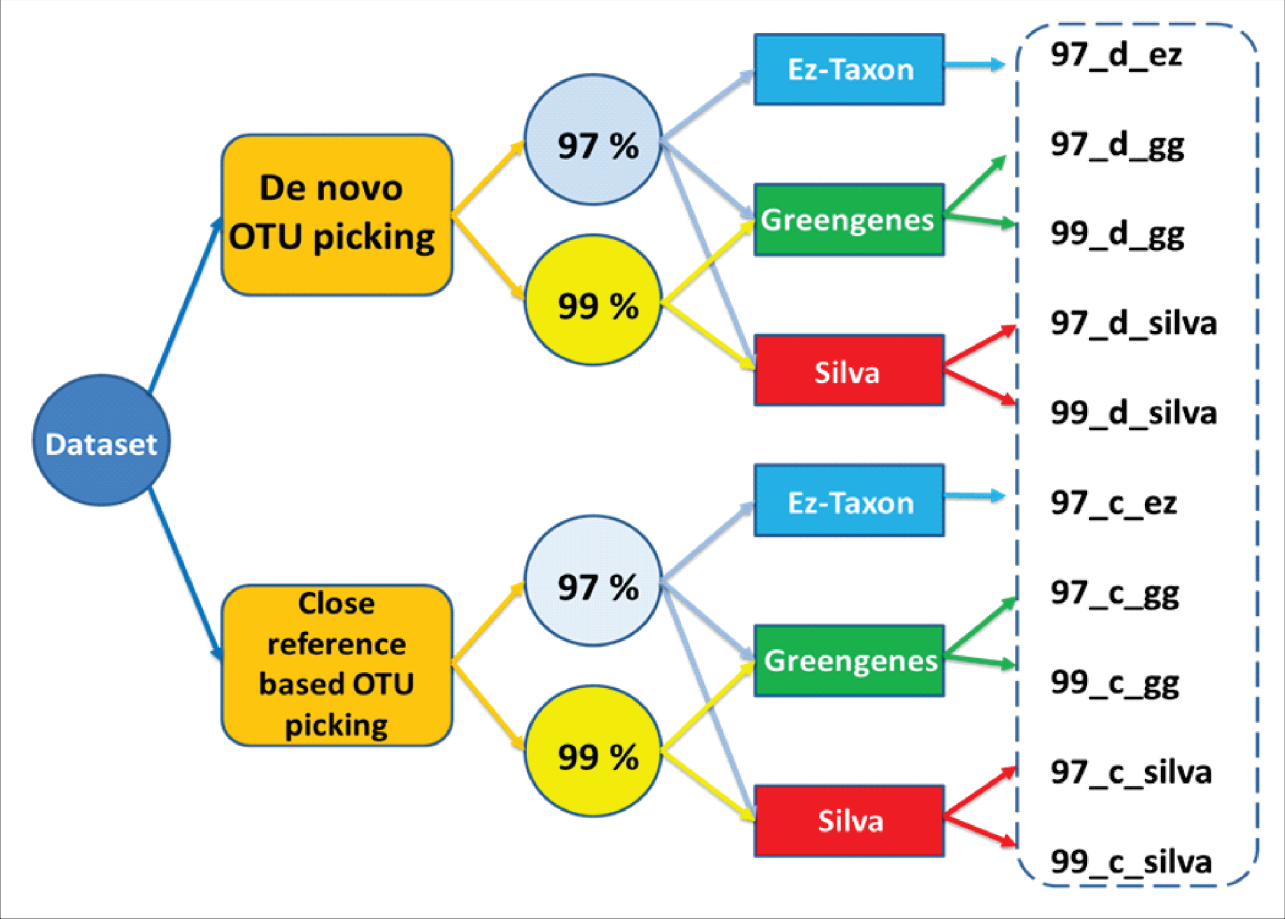
Creation of dataset for analysis

Total 10 different Data Analysis Measures (DAMs) were considered for analysis through the pipeline. They were named as 97_d_ez, 97_d_gg, 97_d_silva, 97_c_ez, 97_c_gg, 97_c_silva, 99_d_gg, 99_d_silva, 99_c_gg, 99_c_silva (Figure 1).

### Selection of data set:

A total of 5895 full-length sequences of 16S rRNA gene along with seven levels of taxonomy were obtained from the RDP official website. Only good quality type strain bacterial sequences with the sequence length >1200 BP were selected were stored in 'fasta' format and used for further analysis.

### Generation of Partial (V3-V4 region) from full-length sequences:

16S rRNA gene sequences were taken from the dataset. 16S rRNA gene sequences show high length variability; hence, to extract the V3-V4 region from the sequences, fuzznuc (from EMBOSS software suite) was used. It extracts the sub-sequence based on PROSITE-style patterns in nucleotide sequences. The primer set used for fuzznuc, CCTACGGGAGGCAGCAG, and GGACTAC [ACT][ACG] GGGT [AT] TCTAAT extracted V3-V4 regions from 91.21% sequences. Fuzznuc failed to extract partial sequences from the remaining 8.79% sequences. These sequences were then manually trimmed using Mega 7 [38].

### Analysis of full length and Partial (V3-V4 region) 16S rRNA gene sequences:

**Figure 2:**
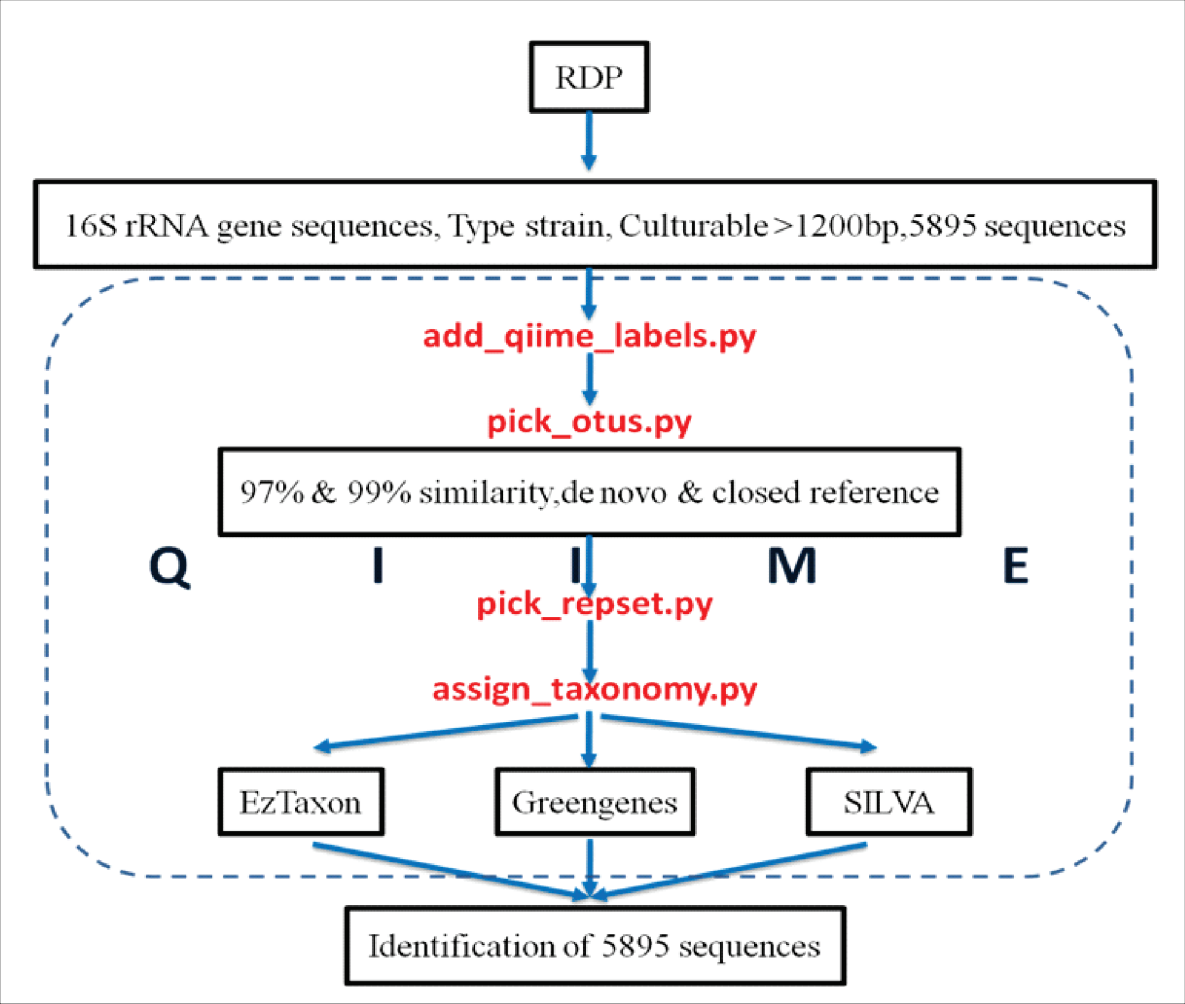
Workflow for steps used in the QIIME pipeline

The open-source data analysis pipeline QIIMEv.1.8 was used for most of the core analyses. Systematic QIIME analysis protocol for analysis included, assignment of valid QIIME labels followed by Operational Taxonomic Units (OTUs) clustering with pre-decided parameters. Further, representative sequence was selected from each OTU and finally taxonomic assignment was done using three databases. OTUs were clustered using two approaches. First, using the de_novo OTU picking method which detects OTUs without comparing the query sequence to any reference. Second, using an Closed reference-based clustering approach. Also, two different identity thresholds i.e., 97% and 99%, were used for OTUs clustering. Figure 2 depicts the flow of methodology used for sequence analysis. Taxonomic identification of sequences was made using three different databases, in combination with OTU clustering method and identity thresholds, and total of ten different DAMs were created for analysis. Further diversity analysis was performed using OTUs table in 'biom' format.

## Results:

### Total OTUs:

**Figure 3:**
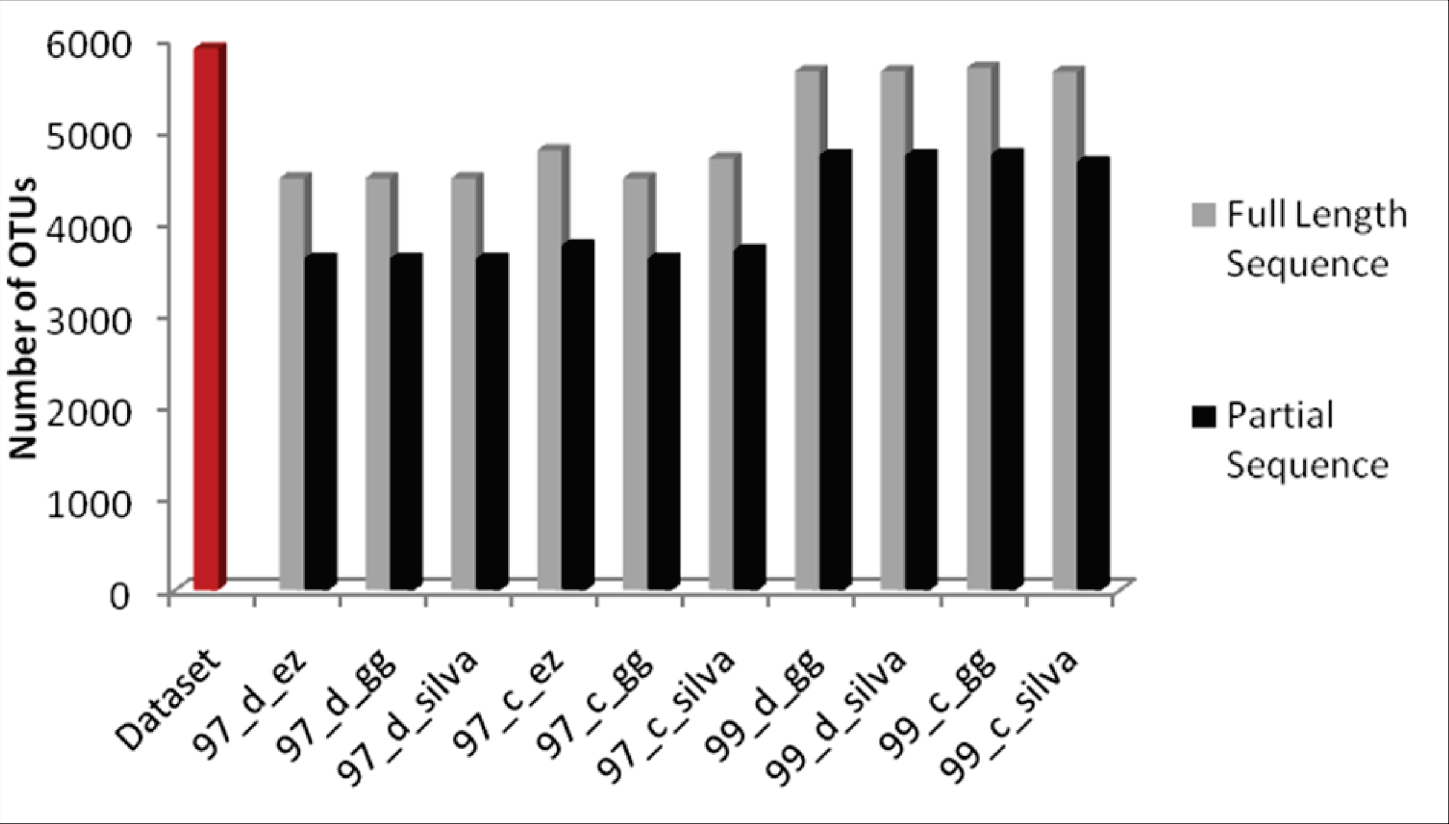
Total number of OTUs observed compared with dataset OTUs

The sample dataset used in this study was obtained from an authentic database, and the sample size, i.e., the number of 16S rRNA gene sequences was 5,895. Also, they were approximately full length (>1200bp) type strain sequences. Considering the parameters mentioned above, it was expected that number of OTUs in the DAMs would be similar to the sample data set. However, it was observed that even with the 97% and 99% identity limits in OTUs picking, the number of OTUs obtained in each variation is significantly different. The total number of OTUs detected in different DAMs is represented in Table 1. Total numbers of OTUs obtained from each DAM were compared; which were less than the Operational Taxonomic Units (number of sequences) in data set (Figure 3). Comparative analysis showed that higher numbers of OTUs were obtained for a 99% identity threshold compared to the 97% identity threshold for the respective combination of the database used. The actual numbers of sequences used for the analysis were 5895. When clustered with the de novo clustering method with 97% and 99% sequence identity thresholds, 23.9% and 4.15% of the total sequences failed to form separate clusters, respectively. In the case of reference-based OTU clustering, EzTaxon, Greengenes, and SILVA databases showed 18.72%, 23.9%, and 20.29% true negatives, respectively, at 97% identity threshold. Whereas at 99% identity threshold value, the percentage of true negatives was reduced to 3.53% and 4.28% for Greengenes and SILVA database, respectively. Partial sequences had a higher amount of true negatives. The de novo OTU clustering method showed 38.77% and 19.72% true negatives for 97% and 99% identity threshold, respectively. Reference-based OTU clustering, EzTaxon, Greengenes, and SILVA databases showed 36.53%, 38.77% and 37.25% true negatives respectively at 97% identity threshold. However, 19.55% and 20.93% true negatives were obtained with a 99% identity threshold for Greengenes and SILVA database, respectively. Highest numbers of OTUs 5,687 (96.47%) were obtained from 99_c_gg and lowest number of OTUs 4,486 (76.09%) from 97_d_ez, 97_d_gg, 97_d_silva and 97_c_gg for full-length sequences. However in case of partial sequences, highest number OTUs obtained were 4743 (80.45%) form 99_c_gg however lowest number of OTUs obtained were 3609 (61.22%) from 97_d_ez, 97_d_gg, 97_d_silva and 97_c_gg (Table 1).

### Overall Classification:

**Figure 4:**
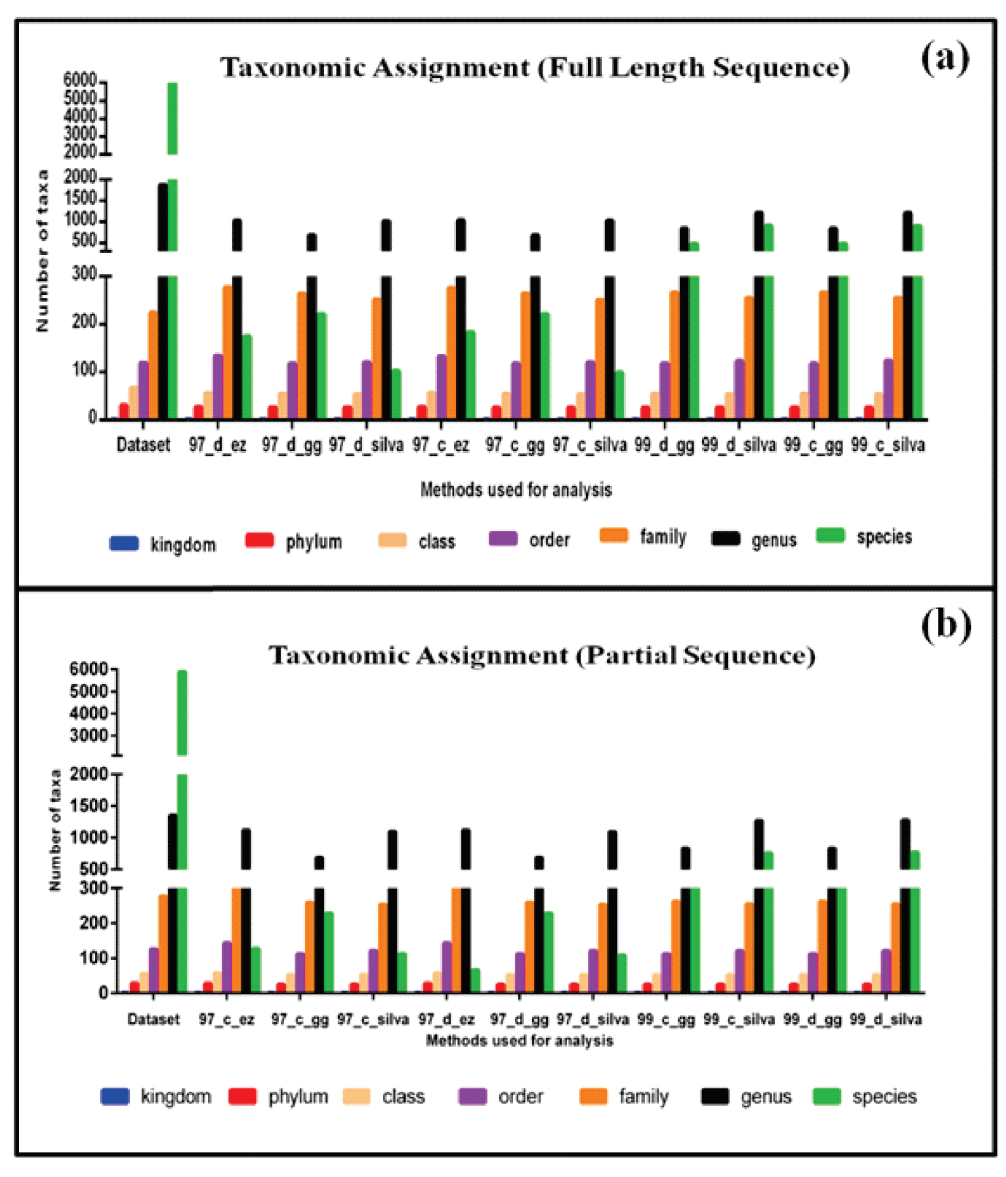
Number of taxonomic assignments obtained for full-length sequences at each taxonomic hierarchy for (a) Full-length sequence and (b) Partial sequence.

In the original data set, the numbers of different taxa were 1, 29, 56, 126, 277, 1422, and 5895 at the kingdom, phylum, class, order, family, genus, and species, respectively. However, in comparison to the original data set, relatively fewer taxa were detected using different DAMs. Also, differences were observed among the DAMs in the total number of taxa detected at different taxonomic levels (Figure 4a-Figure 4b. The graph shows the taxonomic assignments performed with the help of QIIME for the data set using different databases. Original seven-level taxonomy for the data set consisted of 29 phyla. Although taxonomic assignment at the phylum level was observed for most of the sequences using all three databases, not all 30 phyla were observed. Classification by EzTaxon database gave rise to 27 phyla, whereas Greengenes and SILVA databases could classify 26 phyla each for the data set. A total of 1422 genera were present in the data set. SILVA and EzTaxon databases assigned more than 1000 genera in each DAM, but the third database Greengenes could assign less than 1000 genera for its all 4 DAMs. It was observed that no database could successfully assign species-level taxonomy for all the sequences. One possible explanation can be the presence of incomplete taxonomy data present in databases. Furthermore, it was observed that different taxonomic assignment is observed for the same sequences using different pipelines. Thus the same data was checked for the total number of discrepancies present in taxonomic assignments in between different DAMs. Here taxonomy from the original data set was used as a standard control, and discrepancies by each DAM and databases were compared with reference data set.

### Discrepancies:

**Figure 5:**
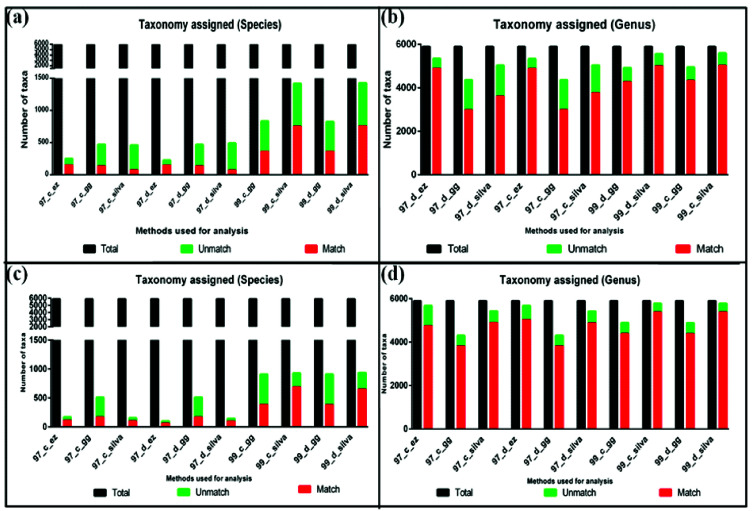
Sequences getting taxonomic assignment as compared with the total number of sequences for (a) species level and (b) genus level for full-length sequences, (c) species level, and (d) genus level for Partial sequences.

**Figure 6:**
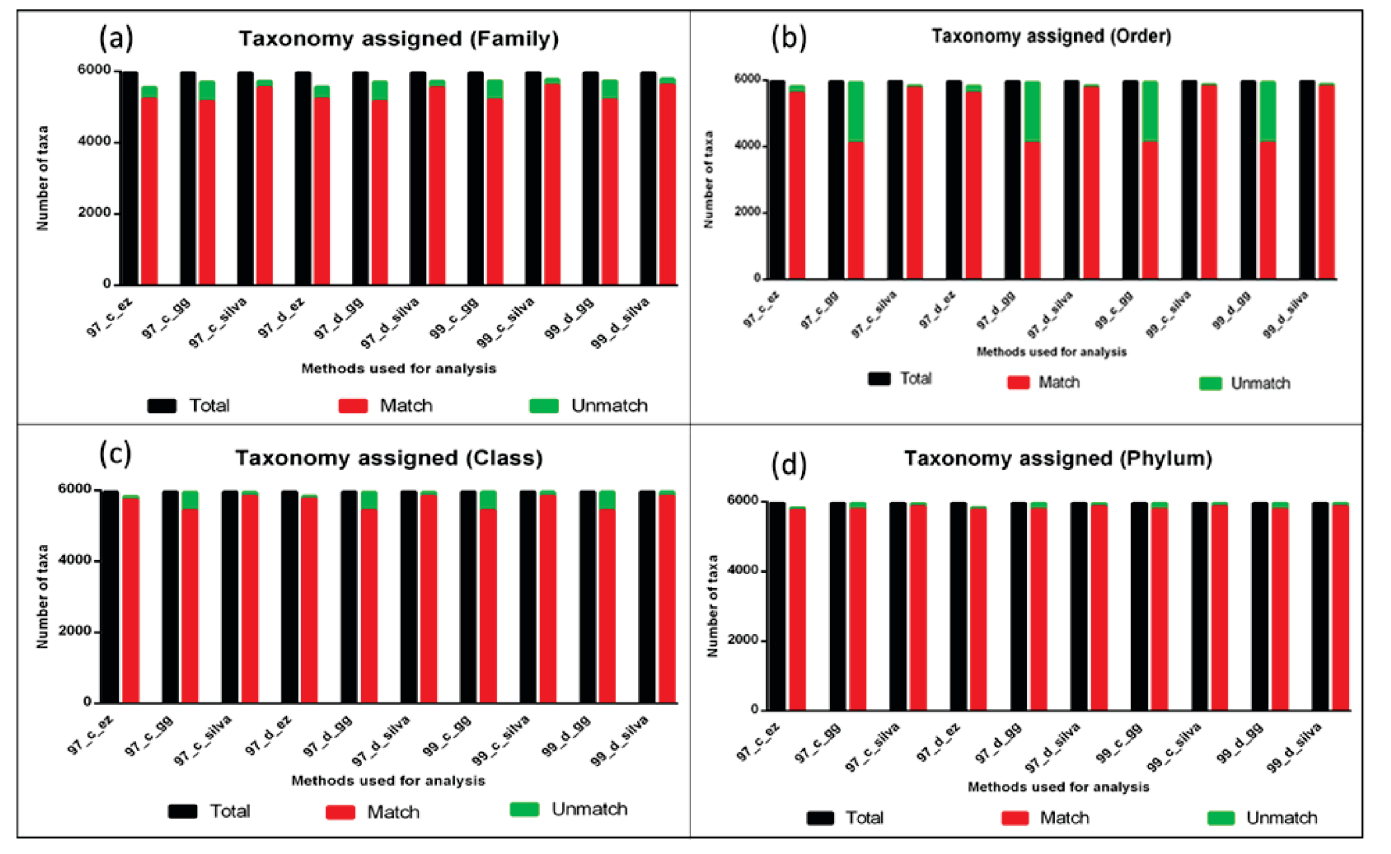
Sequences getting taxonomic assignment at family level (a), Order level (b), Class level (c) and Phylum level (d) compared with total number of sequences for full-length sequences.

**Figure 7:**
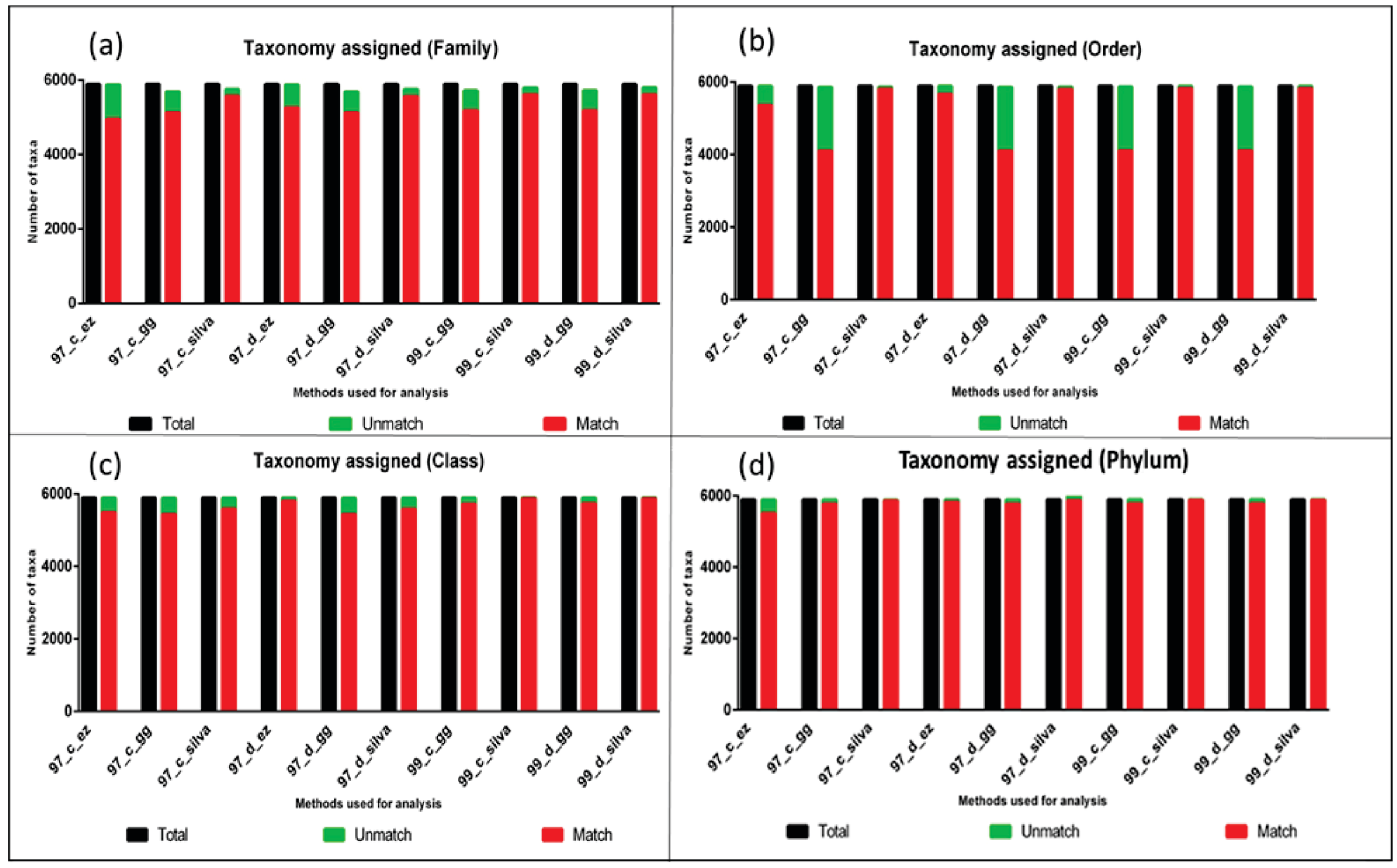
Sequences getting taxonomic assignment at family level (a), Order level (b), Class level (c) and Phylum level (d) compared with total number of sequences for V3-V4 partial sequences.

Incorrect taxonomic classification of a particular type strain sequence using DAMs is referred to as discrepancy in the taxonomic assignment. High amount of discrepancy was observed in the identification of the data set (Figure 5). Percentage discrepancy decreases with higher taxonomy hierarchy. Also, a significantly high amount of discrepancy was observed in the data after analysis through QIIME. A total of 18.78% and 10.53% discrepancy was observed in the identification of type strain data set for the full length and partial sequences, respectively. A discrepancy in the taxonomic assignment was calculated at all taxonomic hierarchies for both full length (Figure 6) and partial sequences (Figure 7). The detailed DAM vise information about classification and discrepancy data for different taxonomic level identifications is represented for full length sequences (Table 2-Table 5) and partial sequences (Table 6 to Table 9).

### Beta Diversity:

**Figure 8:**
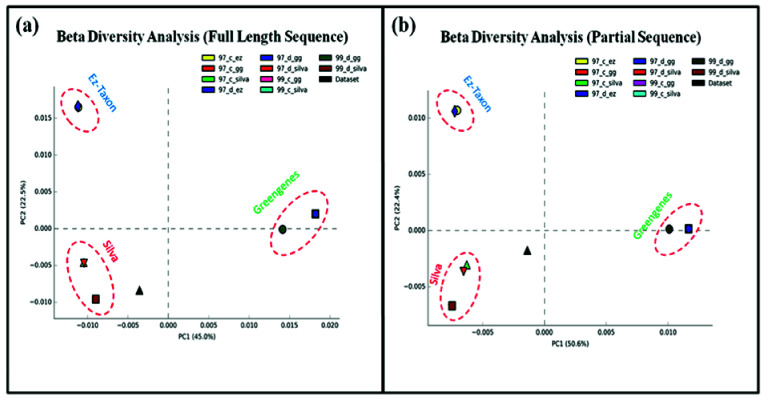
Beta diversity analysis comparing variation in taxonomic assignment by DAMs with reference data set for (a) Full-length sequences and (b) Partial sequences.

Principle Component Analysis (PCA) graphs were plotted for understanding the variation between different samples. A significant variation was observed between databases and standard datasets. Each of the three databases formed their separate clusters in plots for full length as well as partial sequences. Clusters included their variations like 97% and 99% identity thresholds along with de novo and closed reference-based OTU picking method. The original dataset stands alone separately, and some variation exists between data set and clusters. In the case of both full length and partial sequences, SILVA database assigned taxonomy appears closest to the dataset as compared to Greengenes and EzTaxon (Figure 8).

### Misclassification:

Percentage misclassification was varying depending upon the method of picking OTU, identity threshold as well as databases used for the identification. Misclassification was observed using different DAMs at genus level (Table 10). It was observed that results for identification vary with different pipelines. The average misclassification values at genus level were 17.94%. For different DAMs observed misclassification values were 8.67 (97_d_ez), 31.56 (97_d_gg), 28.26 (97_d_silva), 8.49 (97_c_ez), 31.58 (97_c_gg), 25.12 (97_c_silva), 13.1 (99_d_gg), 10.05 (99_d_silva), 12.58 (99_c_gg) and 10.03 (99_c_silva). A total of 48 genera belonging to 26 families were analyzed separately for taxonomic assignment discrepancies. These 48 genera were the most abundant and important in global microbiome studies. Higher misclassification observed in these genera and respective families is evidence of the necessity for correction and up-gradation of databases and software used for bacterial identification using 16S rRNA gene sequencing. Table 11 and Table 12 provide with a list of genera and families used for the top 48 discrepancy analysis, respectively. Table 13 depicts percentage misclassification observed at genus and family level for major human microbiome taxa using different DAMs (Table 13).

## Discussion:

Advances in sequencing technologies have reduced the sequencing cost. Also an increase in computational power has facilitated an overwhelming number of microbiome studies in the diverse ecological niches. A primary goal of all microbiome studies is to identify the bacteria that constitute these complex communities. A valid and reliable method is a must for understanding these complex communities. The purpose of this study was to validate widely used databases like EzTaxon, SILVA, Greengenes, and data analysis pipeline QIIME. Since NGS is becoming cost-effective nowadays, researchers prefer sequencing 16S rRNA gene-based amplicon sequencing for the identification of isolates. The data obtained from sequencing is compared with standard sequences from various databases. In this study, we present results of discrepancies in taxonomic assignment occurred by different databases using the same set of sequences and analyzed through QIIME. Detailed literature review was performed for the selection of QIIME 1.8 as analysis pipeline. We narrowed down to QIIME 1 as it is one of the most used and well-accepted analysis pipeline for microbiome analysis having over 22000 citations. QIIME 1 provides with the OTU selection thresholds of 97%, which is the gold standard identity threshold for 16S rRNA gene analysis for several years. Also, We included 99% identity threshold for comparison purpose. We used data from the RDP database, which provides with the option of bulk data download and is scientifically well-accepted database for the genomic data. Using both full-length sequences (>1200 bp) and partial sequences (V3-V4) of type strain data eliminates chances of bias of selecting only a specific region of 16S rRNA gene and analyzing certain specific sequence data. One of the earlier studies has majorly focused on analyzing data using seven variable regions but has used four mock samples, amplified using the metagenomic kit and a single database[39]. Two other studies used specific variable regions in the 16S rRNA gene for identification [40,41]. Study by Whelan et al. focused on, developing new pipeline which was based on combinations of present computational tools for better reproducibility and visualization of amplicon data analysis [42]. Report by Allali et al. has compared chicken cecum microbiome analysis using different sequencing platforms as well as analysis pipelines proving that the sequencing platform as well as analysis parameters impact microbiome data structure [43]. Carlos et al. spoke about various issues in 16S rRNA gene based analysis regarding assembly, use of short reads and classification inaccuracies [44]. However, there is limited material available validating existing pipelines and tools. To our knowledge, no comparable studies have used full-length 16S rRNA gene sequences analyzed through QIIME with two different OTU picking methods, both with two different identity threshold (97% and 99%) and also using three different widely used databases for the taxonomic assignment. Initially, we have taken 5895 sequences in our data set and used them for OTU picking in QIIME using de novo and closed reference-based methods. Our first results show a number of OTUs obtained after each method. The initial table displays results of OTUs picked by each DAM in comparison with the dataset. Pick_otus.py command classifies closely related individuals in a single cluster. Sequences that show lesser identity than a provided threshold (97% and 99%) are clustered separately as next OTUs. Although the lowest identity threshold provided by us was 97%, the highest numbers of OTUs observed were 5687 for identity threshold of 99%. This suggests that the actual percentage identity between several type strain sequences is more than 97%, thus supporting the newly accepted identity threshold of 98.5% for species designation. This could result in failure of depicting the actual diversity of the sample system. Our aim was to compare two different 16S rRNA gene identity values as several microbiome researchers across the globe use earlier 97% identity threshold as a gold standard [24,25,36, 45,46]. In addition to this, different numbers of assignments were observed at each taxonomic level. The original data set contained 29 phyla, but no DAM showed an assignment of 29 phyla. The highest number of phyla assigned by any DAM was 27, and some identified only 26 phyla. The rest of the sequences either remained unclassified or got misclassified into those identified phyla. Also, during taxonomic assignments using different databases, many of the OTUs remained 'unclassified' at various taxonomic levels. Tables 2-9 provide with a number of OTUs observed to remain unclassified at each taxonomic level for full length as well as partial sequences. The reason behind this is the unavailability of taxonomy for the sequence at that particular taxonomic level in respective databases. Such 'Unclassified' incidences were seen to reduce as we went to higher taxonomic levels, but such occurrences could indicate incompleteness of databases at various taxonomic levels. Such incompleteness of databases might fail to provide the taxonomic identification till species level. Another important observation was noted about misclassification and discrepancy in taxonomic assignment. Taxonomy assigned for the sequence by QIIME pipeline with a specific database was completely different from the original taxonomy in the data set. Varying discrepancies were observed in assigned taxonomy at each taxonomic level and in each of the 10 DAMs. A total of 18.78% discrepancy in assigned taxonomy was observed at the genus level, which is a high amount of discrepancy. Genus level classification is mentioned here because genus and species are two taxonomic hierarchies that we generally use for referring any organism. The percentage of discrepancy was seen to reduce with higher taxonomic hierarchy. Table 10 enlists some of the misclassified organisms. Each database showed a different percentage of discrepancy in the taxonomic assignment possibly due to different time intervals of database update or the methodological limitations of the analysis measures. The highest discrepancy was observed as 31.58% for 97_c_gg and lowest discrepancy was observed as 4.37% for 97_d_ez at the genus level. Such misclassification i.e., assignment of different taxonomies for the same sequences by different databases, makes it difficult to choose a suitable database for analysis purposes. Generally, only single pipeline and database is used for any analysis and identification purpose by any researcher or sequencing facilities or even pathology labs [3] for identification of the pathogen. Such improper identifications could hamper analysis reliability. In recent years, the field of microbiome research has moved ahead from just exploring the microbial diversity in the sample to interventional and translational research. Hence, it is crucial to use correct bacterial strains. The use of wrong bacterial strains can be deleterious. One of the studies clearly states the misclassification issue of genus Acinetobacter with phylogenomic approach. It has now been established that the genus is poorly classified, especially for closely related species like Acinetobacter calcoaceticus and Acinetobacter baumannii complex (Acb complex) [47]. Such inaccurate assignment in taxonomic classifier might possibly lead to community member misclassification and ultimately misleading conclusions and possibly treatment. In this study, we explored most widely used 16S rRNA gene databases and QIIME pipeline with various parameters. A similar approach can be used to benchmark any new pipeline or database before analyzing actual data. The reference set allows us to cross-validate taxonomic assignments. In the last decade, a lot of microbiome data is available for various ecosystems, like soil, water, plant, animal, and human. This has enabled us to get a fair idea about the taxonomic groups expected in the ecosystem. A combination of a particular pipeline and database can be selected based on the accuracy in assignment taxa expected in the ecosystem. This study gives a fair idea about the limitation of using short reads for taxonomic assignment as well as about the list of important taxa that can be misclassified using a particular analysis pipeline.

## Conclusion

99% identity threshold is better for OTU clustering for full length as well as partial length sequences than conventional 97% identity threshold. A total 18.78% and 10.53% discrepancy was observed at the genus level for the full length and partial sequences, respectively, which is a high amount of discrepancy. The discrepancy at each taxonomic level can be calculated, and the quality of data present in the database can be decided. Beta diversity analysis shows an overall distance of analyzed data from the reference data set. 99_c_silva shows most identity with reference data set as compared to other DAMs. 99_c_silva means SILVA database with a identity threshold of 99% and close reference-based OTU picking method. It is crucial to select databases, pipelines, and algorithms very carefully considering discrepancies in taxonomic assignment and selection should be done based on the necessity of the study. Also, databases should be validated, and discrepancies should be corrected in successive updates of databases.

## Figures and Tables

**Table 1:** Observed number of Operational Taxonomic Units (OTUs) for different DAMs and sequence lengths.

S. no	DAMs	Total OTUs (Full Length)	Total OTUs (Partial)
1	97_d_ez	4486	3609
2	97_d_gg	4486	3609
3	97_d_silva	4486	3609
4	97_c_ez	4789	3752
5	97_c_gg	4486	3609
6	97_c_silva	4699	3699
7	99_d_gg	5650	4733
8	99_d_silva	5650	4733
9	99_c_gg	5687	4743
10	99_c_silva	5643	4661

**Table 2:** Classification and discrepancy data for family level identification for full-length sequences.

DAMs	Total	Unclassified family	Not Assigned	Taxonomy assigned	Classification (%)	Match	Unmatch	Match (%)	Discrepancy (%)
97_d_ez	5895	278	58	5559	94.3	5230	329	94.08	5.92
97_d_gg	5895	180	11	5704	96.76	5164	540	90.53	9.47
97_d_silva	5895	166	8	5721	97.05	5542	179	96.87	3.13
97_c_ez	5895	280	63	5552	94.18	5225	327	94.11	5.89
97_c_gg	5895	180	11	5704	96.76	5164	540	90.53	9.47
97_c_silva	5895	164	7	5724	97.1	5545	179	96.87	3.13
99_d_gg	5895	156	6	5733	97.25	5200	533	90.7	9.3
99_d_silva	5895	102	4	5789	98.2	5617	172	97.03	2.97
99_c_gg	5895	156	6	5733	97.25	5200	533	90.7	9.3
99_c_silva	5895	108	4	5783	98.1	5611	172	97.03	2.97
							Total discrepancy (%)	6.15	

**Table 3:** Classification and discrepancy data for order level identification for full-length sequences.

DAMs	Total	Unclassified order	Not Assigned	Taxonomy assigned	Classification (%)	Match	Unmatch	Match (%)	Discrepancy (%)
97_d_ez	5895	22	58	5815	98.64	5623	192	96.7	3.3
97_d_gg	5895	26	11	5858	99.37	4112	1746	70.19	29.81
97_d_silva	5895	54	8	5833	98.95	5771	62	98.94	1.06
97_c_ez	5895	24	63	5808	98.52	5619	189	96.75	3.25
97_c_gg	5895	26	11	5858	99.37	4112	1746	70.19	29.81
97_c_silva	5895	58	7	5830	98.9	5769	61	98.95	1.05
99_d_gg	5895	24	6	5865	99.49	4116	1749	70.18	29.82
99_d_silva	5895	11	4	5880	99.75	5814	66	98.88	1.12
99_c_gg	5895	24	6	5865	99.49	4116	1749	70.18	29.82
99_c_silva	5895	18	4	5873	99.63	5807	66	98.88	1.12
							Total discrepancy (%)	13.02	

**Table 4:** Classification and discrepancy data for class level identification for full-length sequences.

DAMs	Total	Unclassified class	Not Assigned	Taxonomy assigned	Classification (%)	Match	Unmatch	Match (%)	Discrepancy (%)
97_d_ez	5895	2	58	5835	98.98	5767	68	98.83	1.17
97_d_gg	5895	5	11	5879	99.73	5440	439	92.53	7.47
97_d_silva	5895	7	8	5880	99.75	5838	42	99.29	0.71
97_c_ez	5895	2	63	5830	98.9	5741	89	98.47	1.53
97_c_gg	5895	5	11	5879	99.73	5440	439	92.53	7.47
97_c_silva	5895	11	7	5877	99.69	5836	41	99.3	0.7
99_d_gg	5895	5	6	5884	99.81	5444	440	92.52	7.48
99_d_silva	5895	4	4	5887	99.86	5842	45	99.24	0.76
99_c_gg	5895	5	6	5884	99.81	5444	440	92.52	7.48
99_c_silva	5895	4	4	5887	99.86	5842	45	99.24	0.76
							Total discrepancy (%)	3.55	

**Table 5:** Classification and discrepancy data for phylum level identification for full-length sequences.

DAMs	Total	Unclassified phylum	Not Assigned	Taxonomy assigned	Classification (%)	Match	Unmatch	Match (%)	Discrepancy (%)
97_d_ez	5895	2	58	5835	98.98	5769	66	98.87	1.13
97_d_gg	5895	0	11	5884	99.81	5776	108	98.16	1.84
97_d_silva	5895	6	8	5881	99.76	5868	13	99.78	0.22
97_c_ez	5895	2	63	5830	98.9	5764	66	98.87	1.13
97_c_gg	5895	0	11	5884	99.81	5776	108	98.16	1.84
97_c_silva	5895	10	7	5878	99.71	5866	12	99.8	0.2
99_d_gg	5895	0	6	5889	99.9	5781	108	98.17	1.83
99_d_silva	5895	3	4	5888	99.88	5872	16	99.73	0.27
99_c_gg	5895	0	6	5889	99.9	5781	108	98.17	1.83
99_c_silva	5895	3	4	5888	99.88	5872	16	99.73	0.27
							Total discrepancy (%)	1.06	

**Table 6:** Classification and discrepancy data for family level identification for partial sequences.

DAMs	Total	Unclassified family	Not Assigned	Taxonomy assigned	Classification (%)	Match	Unmatch	Match (%)	Discrepancy (%)
97_c_ez	5894	13	0	5881	99.78	4960	921	84.34	15.66
97_c_gg	5894	204	0	5690	96.54	5134	556	90.23	9.77
97_c_silva	5894	137	0	5757	97.68	5577	180	96.87	3.13
97_d_ez	5894	14	0	5880	99.76	5267	613	89.57	10.43
97_d_gg	5894	204	0	5690	96.54	5134	556	90.23	9.77
97_d_silva	5894	139	0	5755	97.64	5570	185	96.79	3.21
99_c_gg	5894	169	0	5725	97.13	5190	535	90.66	9.34
99_c_silva	5894	99	0	5795	98.32	5617	178	96.93	3.07
99_d_gg	5894	170	0	5724	97.12	5188	536	90.64	9.36
99_d_silva	5894	96	0	5798	98.37	5609	189	96.74	3.26
							Total discrepancy (%)	7.7	

**Table 7:** Classification and discrepancy data for order level identification for partial sequences.

DAMs	Total	Unclassified order	Not Assigned	Taxonomy assigned	Classification (%)	Match	Unmatch	Match (%)	Discrepancy (%)
97_c_ez	5894	6	0	5888	99.9	5365	523	91.12	8.88
97_c_gg	5894	37	0	5857	99.37	4106	1751	70.1	29.9
97_c_silva	5894	32	0	5862	99.46	5805	57	99.03	0.97
97_d_ez	5894	7	0	5887	99.88	5674	213	96.38	3.62
97_d_gg	5894	37	0	5857	99.37	4106	1751	70.1	29.9
97_d_silva	5894	31	0	5863	99.47	5800	63	98.93	1.07
99_c_gg	5894	27	0	5867	99.54	4112	1755	70.09	29.91
99_c_silva	5894	12	0	5882	99.8	5823	59	99	1
99_d_gg	5894	27	0	5867	99.54	4112	1755	70.09	29.91
99_d_silva	5894	12	0	5882	99.8	5823	59	99	1
							Total discrepancy (%)	13.62	

**Table 8:** Classification and discrepancy data for class level identification for partial sequences.

DAMs	Total	Unclassified class	Not Assigned	Taxonomy assigned	Classification (%)	Match	Unmatch	Match (%)	Discrepancy (%)
97_c_ez	5894	5	0	5889	99.92	5489	400	93.21	6.79
97_c_gg	5894	14	0	5880	99.76	5439	441	92.5	7.5
97_c_silva	5894	13	0	5881	99.78	5580	301	94.88	5.12
97_d_ez	5894	6	0	5888	99.9	5798	90	98.47	1.53
97_d_gg	5894	14	0	5880	99.76	5439	441	92.5	7.5
97_d_silva	5894	13	0	5881	99.78	5579	302	94.86	5.14
99_c_gg	5894	8	0	5886	99.86	5722	163	97.21	2.79
99_c_silva	5894	2	0	5892	99.97	5869	23	99.61	0.39
99_d_gg	5894	8	0	5886	99.86	5735	151	97.43	2.57
99_d_silva	5894	2	0	5892	99.97	5869	23	99.61	0.39
							Total discrepancy (%)	3.97	

**Table 9:** Classification and discrepancy data for phylum level identification for partial sequences.

DAMs	Total	Unclassified phylum	Not Assigned	Taxonomy assigned	Classification (%)	Match	Unmatch	Match (%)	Discrepancy (%)
97_c_ez	5894	5	0	5889	99.92	5513	376	93.62	6.38
97_c_gg	5894	9	0	5885	99.85	5776	109	98.15	1.85
97_c_silva	5894	13	0	5881	99.78	5864	17	99.71	0.29
97_d_ez	5894	6	0	5888	99.9	5822	66	98.88	1.12
97_d_gg	5894	9	0	5885	99.85	5776	109	98.15	1.85
97_d_silva	5894	13	0	5881	99.78	5865	516	99.73	0.27
99_c_gg	5894	3	0	5891	99.95	5782	109	98.15	1.85
99_c_silva	5894	2	0	5892	99.97	5877	15	99.75	0.25
99_d_gg	5894	3	0	5891	99.95	5782	109	98.15	1.85
99_d_silva	5894	2	0	5892	99.97	5877	15	99.75	0.25
							Total discrepancy (%)	1.6	

**Table 10:** List of some of the misclassified organisms. Taxonomy assigned by different databases is different from their actual taxonomy in the reference data set.

ID	Actual Classification	ID	Misclassification
53	Pediococcus_acidilactici	53	Pediococcus_lolii
509	Kluyvera_ascorbata	509	Kluyvera_cryocrescens
565	Corynebacterium_aurimucosum	565	Corynebacterium_lubricantis
616	Carnobacteriaceae_bacterium	616	Pisciglobus_halotolerans
764	Salmonella_bongori	764	Salmonella_enterica
790	Moraxella_bovoculi	790	Moraxella_canis
809	Pantoea_brenneri	809	Pantoea_stewartii
811	Bifidobacterium_breve	811	Bifidobacterium_longum
945	Porphyromonas_cansulci	945	Porphyromonas_crevioricanis
947	Streptosporangium_canum	947	Streptosporangium_oxazolinicum
1020	Pseudomonas_cissicola	1020	Xanthomonas_citri
1316	Pantoea_conspicua	1316	Pantoea_stewartii
1369	Arthrobacter_creatinolyticus	1369	Glutamicibacter_creatinolyticus
2222	Mycobacterium_goodii	2222	Mycobacterium_wolinskyi
2316	Haemophilus_haemoglobinophilus	2316	Pasteurella_multocida

**Table 11:** List of top 48 genera in global microbiome studies, which were considered for discrepancy analysis.

Genera analyzed			
1	Acidaminococcus	25	Enterococcus
2	Acinetobacter	26	Faecalibacterium
3	Actinomyces	27	Flavobacterium
4	Adlercreutzia	28	Fusobacterium
5	Akkermansia	29	Haemophilus
6	Anaerostipes	30	Klebsiella
7	Bacillus	31	Lachnospira
8	Bacteroides	32	Lactobacillus
9	Bifidobacterium	33	Megamonas
10	Bilophila	34	Megasphaera
11	Blautia	35	Mitsuokella
12	Bulleidia	36	Odoribacter
13	Butyricimonas	37	Parabacteroides
14	Butyrivibrio	38	Paraprevotella
15	Catenibacterium	39	Plesiomonas
16	Citrobacter	40	Prevotella
17	Clostridium	41	Pseudomonas
18	Collinsella	42	Roseburia
19	Coprobacillus	43	Ruminococcus
20	Coprococcus	44	Slackia
21	Delftia	45	Streptococcus
22	Desulfovibrio	46	Succinivibrio
23	Dialister	47	Sutterella
24	Dorea	48	Veillonella

**Table 12:** List of families taken into consideration for discrepancy analysis. Top 48 genera in global microbiome studies belong to these 26 families.

Families analyzed
1	Actinomycetaceae	14	Fusobacteriaceae
2	Alcaligenaceae	15	Lachnospiraceae
3	Bacillaceae	16	Lactobacillaceae
4	Bacteroidaceae	17	Moraxellaceae
5	Bifidobacteriaceae	18	Pasteurellaceae
6	Clostridiaceae	19	Porphyromonadaceae
7	Comamonadaceae	20	Prevotellaceae
8	Coriobacteriaceae	21	Pseudomonadaceae
9	Desulfovibrionaceae	22	Ruminococcaceae
10	Enterobacteriaceae	23	Streptococcaceae
11	Enterococcaceae	24	Succinivibrionaceae
12	Erysipelotrichaceae	25	Veillonellaceae
13	Flavobacteriaceae	26	Verrucomicrobiaceae

**Table 13:** Percentage Misclassification or no classification in top 48 genera in Human Microbiome using different methods of analysis.

Sequence Length	Method	97_d_ez	97_d_gg	97_d_silva	97_c_ez	97_c_gg	97_c_silva	99_d_gg	99_d_silva	99_c_gg	99_c_silva
Full Length	Genus	97.87	20.05	21.3	94.29	20.05	21.09	14.31	8.49	12.27	9.32
	Family	11.17	6.52	3.99	11.44	6.52	3.97	6.4	3.52	6.4	3.48
Partial	Genus	9.7	11.88	11.38	9.67	11.88	11.29	14.11	3.85	14.09	6.08
	Family	10.1	6.5	3.95	10.12	6.5	3.79	6.57	3.22	6.57	3.22
